# Levels and Trends of Maternal Mortality and Morbidity by Cause in North Africa and Middle East, 1990 to 2019: An Analysis for the Global Burden of Disease Study 2019

**DOI:** 10.34172/aim.2022.105

**Published:** 2022-10-01

**Authors:** Sadaf G. Sepanlou, Hossein Rezaei Aliabadi, Reza Malekzadeh, Mohsen Naghavi, GBD Maternal Middle East Collaborators

**Affiliations:** ^1^Digestive Disease Research Institute, Tehran University of Medical Sciences, Tehran, Iran; ^2^Bam University of Medical Sciences, Bam, Iran; ^3^Institute for Health Metrics and Evaluation, School of Medicine, University of Washington, Seattle, USA

**Keywords:** Maternal mortality, Maternal disorder, Maternal health services, North Africa and Middle East, Global Burden of Disease

## Abstract

**Background:**

Since 1990, the maternal mortality significantly decreased at global scale as well as the North Africa and Middle East. However, estimates for mortality and morbidity by cause and age at national scale in this region are not available.

**Methods:**

This study is part of the Global Burden of Diseases, Injuries, and Risk Factors study (GBD) 2019. Here we report maternal mortality and morbidity by age and cause across 21 countries in the region from 1990 to 2019.

**Results:**

Between 1990 and 2019, maternal mortality ratio (MMR) dropped from 148.8 (129.6–171.2) to 94.3 (73.4–121.1) per 100000 live births in North Africa and Middle East. In 1990, MMR ranged from 6.0 (5.3–6.8) in Kuwait to 502.9 (375.2–655.3) per 100000 live births in Afghanistan. Respective figures for 2019 were 5.1 (4.0–6.4) in Kuwait to 269.9 (195.8–368.6) in Afghanistan. Percentages of deaths under 25 years was 26.0% in 1990 and 23.8% in 2019. Maternal hemorrhage, indirect maternal deaths, and other maternal disorders rank 1^st^ to 3^rd^ in the entire region. Ultimately, there was an evident decrease in MMR along with increase in socio-demographic index from 1990 to 2019 in all countries in the region and an evident convergence across nations.

**Conclusion:**

MMR has significantly declined in the region since 1990 and only five countries (Afghanistan, Sudan, Yemen, Morocco, and Algeria) out of 21 nations didn’t achieve the Sustainable Development Goal (SDG) target of 70 deaths per 100000 live births in 2019. Despite the convergence in trends, there are still disparities across countries.

## Introduction

 Worldwide, during the past four decades, there has been increasing focus on reducing maternal mortality and morbidity. In 1987 the safe motherhood initiative was launched by the United Nations Agencies to reduce maternal mortality worldwide.^1^ In 2003, improvement in maternal health was set by the United Nations as the 5^th^ Millennium Development Goal (MDGs) to reduce maternal mortality ratio (MMR) by three quarters until 2015.^2^ In September 2005 the Partnership for Maternal, Newborn and Child Health was established in the World Health Organization (WHO) to enhance the achievements stated in MDGs.^3^ Ultimately, in 2015, the United Nations announced 17 Sustainable Development Goals (SDGs), one of which was focused on maternal mortality.^4^ The target was to decline MMR to less than 70 per 100 000 live births by 2030. Overall, we observe a substantial global decline in MMR during the past 30 years. Based on recent estimates, between 2000 and 2017, the MMR dropped by 38% worldwide, from 342 deaths to 211 deaths per 100 000 live births, while 94% of all maternal deaths occur in low and middle income countries.^5^ Although the average annual reduction rate of 2.9% seems to be substantive, it is not adequate to achieve the SDG target by 2030 at global scale. The average annual reduction should be more than 6.4% to achieve the target.

 Countries in North Africa and Middle East have also made tremendous progress in reducing maternal mortality since 1990. The MMR declined by 50% in the region from 1990 to 2015 (from 220 to 110 maternal deaths per 100 000 live births).^6^ However, there is still substantial heterogeneity between countries in the region in terms of socio-economic status, development status, and subsequently maternal mortality and morbidity.^6^ While high-income countries in the region take advantage of their infrastructure to provide high-quality care for mothers and children, low-income countries suffer from deficiencies in infrastructure, logistics, funds, and human resources.^5^ A number of nations are additionally afflicted by war, conflicts, and social and economic insecurity, which has led to the disruption of their health systems.^7^

 Specific estimates for maternal mortality in North Africa and Middle East at regional and national level were released by UNICEF in 2015.^6^ Global, regional, and national estimates for maternal mortality were published by the UN Maternal Mortality Estimation Inter-Agency Group in 2016.^8^ Ultimately, in 2019 WHO released trends in maternal mortality from 2000 to 2017.^5^ Similar estimates were made by the Global Burden of Diseases, Injuries, and Risk Factors (GBD) study 2013 and 2017.^9-15^ To the best of our knowledge, these sources have provided the most comprehensive estimates for maternal mortality in the region so far. However, in the current and most recent version of GBD 2019, the estimates have been made for both maternal mortality and morbidity, by cause and by age, from 1990 to 2019, and across 21 countries in the region.

## Materials and Methods

 This study was part of GBD 2019, which was a systematic effort to estimate the levels, trends, and causes of mortality and morbidity by sex, age, year (1990 to 2019), and location.^16^ In this article we report estimates for fatal and non-fatal maternal disorders in North Africa and Middle East and across 21 countries including: Afghanistan, Algeria, Bahrain, Egypt, Iran, Iraq, Jordan, Kuwait, Lebanon, Libya, Morocco, Oman, Palestine, Qatar, Saudi Arabia, Sudan, Syrian Arab Republic, Tunisia, Turkey, United Arab Emirates, and Yemen. Over 650 million people live in this region, including 185 million adolescent and young adults aged between 10 and 24 years (28% of the population).^10^

 To estimate overall and cause-specific maternal mortality and morbidity, we used data from vital registrations, death registration systems, maternal mortality surveillance systems, censuses, Maternal Mortality Reports, results of Reproductive Age Mortality Studies (RAMOS), Integrated Micronutrient Surveys, National Health Accounts, Multiple Indicator Cluster Surveys (MICS), and the published scientific literature on maternal mortality and disorders in North Africa and Middle East. Our systematic literature review for maternal disorders is updated annually and encompasses all aspects of maternal disorder burden estimation.^9,10^

###  Maternal Mortality

 There has been much debate about the definition of maternal deaths. To be classified as maternal, pregnancy needs to be a causal factor in death. It can either have a direct effect (complications of the pregnancy or childbirth, or postpartum complications) or indirect effect (exacerbation of a pre-existing condition). Therefore, accidental or incidental deaths in which pregnancy had no causal role are not classified as maternal deaths.^11^ We included direct and indirect deaths during pregnancy and within 6 weeks of delivery, plus late maternal deaths after 6 weeks up to 1 year after delivery and the fraction of HIV-related deaths aggravated by pregnancy.^11^ We disaggregated maternal deaths into ten causes: 1) maternal hemorrhage, 2) maternal sepsis and other pregnancy-related infections, 3) hypertensive disorders of pregnancy, 4) obstructed labor, 5) abortion, 6) other direct maternal disorders, 7) indirect maternal disorders, 8) ectopic pregnancy, 9) HIV, and 10) late maternal deaths.^9^

 For overall maternal mortality and cause-specific mortality, all data were reviewed in cause of death ensemble models (CODEm). The details are previously published.^17^ Covariates included in the model for overall maternal mortality, their level, and directionality are show in Table S1 (Supplementary file 1). Outliers were identified as those data where age patterns or temporal patterns were inconsistent with neighboring age groups or locations or where sparse data were predicting implausible overall temporal or age patterns for a given location. All cause-specific maternal mortality data were extracted as maternal mortality ratio (MMR; cause-specific deaths per live births). All cause of death (COD) data, along with any sources that reported cause-specific maternal deaths in cause fraction or population rate terms, were converted to MMR using all-cause mortality, population, and age-specific fertility results estimated in GBD 2019.^9,10^ We used spatiotemporal Gaussian process regression (ST-GPR) to estimate MMRs for each of the maternal sub-causes.^9,10^ Covariates are demonstrated in Table S2 (Supplementary file 1).

 Cause-specific estimates were derived by scaling the results from the ST-GPR subcause-specific

 models scaled in relation to each other to equal one and then multiplying them by the total maternal deaths, corrected for late maternal deaths, for that age group, location, and year. A single parameter proportion model was run in DisMod-MR 2.1, which is a Bayesian meta-regression tool developed for the GBD, for late maternal deaths using the data described above. The final result includes cause fraction and number of maternal deaths due to each cause, by country and province, age group, and year. All cause-specific MMR and proportion data were uploaded to the non-fatal database.^9,10^

###  Maternal Morbidity

 Maternal disorders nonfatal estimation includes disability due to seven of ten maternal mortality sub-causes, excluding indirect maternal deaths, late maternal deaths, and maternal deaths aggravated by HIV/AIDS, which did not have any estimated disability.^9,10^

 All data were either extracted as incidence ratio (number of events / live birth) or, if data were only available with population as the denominator, they were converted to incidence ratio using GBD 2019 age-specific fertility rate (number of live births/population). The reason is that most literature and surveillance data are expressed in terms of number of events per live birth rather than per population. Hospital and claims data, which were centrally processed for all GBD 2019 causes to have population as the denominator, were transformed to have livebirths as the denominator by dividing by age-specific fertility rate (ASFR; live births per population).

 The first step of data processing was age splitting. For any datum that did not entirely fit within a GBD age group, the observation was split to be multiple age-specific data points based on the age pattern predicted by GBD 2017 DisMod-MR 2.1 models. It is our intention to update this age splitting with each cycle of GBD. The second step was cross-walking all data from alternate to reference definitions. We adjusted data to the reference category for each cause by age using MR-BRT (meta regression-Bayesian, regularized, trimmed), a meta-analytic tool developed for GBD 2019. The details of each of the crosswalks are previously published.^9,10^ All data sources that only reported event rates for severe maternal morbidity or “near miss” were excluded as a reliable crosswalk model could not be developed.

 We estimated the incidence ratio of each category of pregnancy complications for each age-location-year in the GBD 2019 location hierarchy using DisMod-MR 2.1. After completion of DisMod-MR 2.1 models, all age-specific ratios were then converted to incidence rates by multiplying by ASFR and then to prevalence rates by applying a global assumed duration of disability for each type of pregnancy complications.^9,10^ We quantified disability weights for each maternal disorder and finally calculated years lived with disability (YLD) for each 7 maternal disorders. Disability-adjusted life years (DALYs) were the sum of YLDs and years lost due to premature death (YLLs) previously estimated for maternal mortality.^9,10^

 We used the socio-demographic index (SDI) to determine the relationship between the development level of a province and maternal mortality ratio. In GBD 2017, the SDI was revised to better reflect the development status of countries and provinces. The SDI ranges from 0 (worst) to 1 (best) and is a composite measure of the total fertility rate in women under the age of 25 years, mean education for individuals aged 15 years and older, and lag-distributed income per capita.^12-14^ We report 95% uncertainty intervals (UIs) for all estimates. All-cause and cause-specific mortality and morbidity estimation components are based on 1000 draws, or simulations, by age, sex, location, and year. Point estimates were derived from the mean of the draws, and 95% UIs were calculated as the 2.5^th^ and 97.5^th^ percentiles of the draws.

## Results

**Figure 1:**
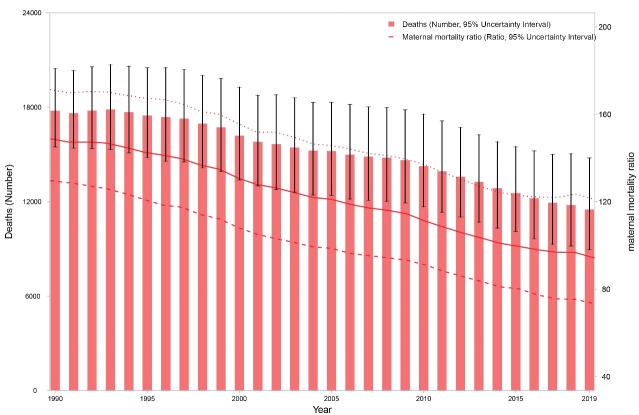
Number of Maternal Deaths and Maternal Mortality Ratio from 1990 to 2019 in North Africa and Middle East.

 In 1990, 17 779 (95% UI: 15 488-20 461) deaths occurred due maternal disorders in North Africa and Middle East, which decreased to 11 505 (8953–14 775) deaths in 2019. During this time period, MMR dropped from 148.8 (129.6–171.2) to 94.3 (73.4–121.1) per 100 000 live births (Table 1 and Figure 1). Meanwhile, the number of DALYs were 1 120 074 (986 199-1 275 046) years in 1990 and 740 462 (593 259–930 918) years in 2019. Respective figures for age-standardized DALY rates were 715.6 (630.4–817.7) and 228.8 (183.7–287.7) per 100 000 females (Figure S1, Table S3).

**Table 1:** Maternal Deaths and Maternal Mortality Ratio Per 100 000 Live Births Across Countries in North Africa and Middle East and the Percent Change in Ratios from 1990 to 2019

	**Maternal Deaths 1990 ** **(95% UI)**	**MMR 1990 ** **(95% UI)**	**Maternal Deaths 2019 (95% UI)**	**MMR 2019 ** **(95% UI)**	**Percent Change in Ratios**
North Africa and Middle East	17779 (15488, 20461)	148.8 (129.6, 171.2)	11505 (8953, 14775)	94.3 (73.4, 121.1)	-36.6 (-49.3, -19.7)
Afghanistan	2655 (1981, 3460)	502.9 (375.2, 655.3)	4038 (2930, 5514)	269.9 (195.8, 368.6)	-46.3 (-61.1, -23)
Algeria	1634 (1221, 2149)	212.2 (158.5, 279.1)	638 (483, 833)	72 (54.5, 94)	-66.1 (-76.6, -49.8)
Bahrain	6 (4, 7)	40.5 (32.5, 49.5)	5 (4, 6)	38.4 (29.2, 49.9)	-5.2 (-31.7, 33)
Egypt	2041 (1780, 2316)	104.4 (91.1, 118.5)	751 (514, 1056)	35.7 (24.4, 50.1)	-65.9 (-76.8, -52.1)
Iran (Islamic Republic of)	776 (674, 875)	44.5 (38.6, 50.1)	214 (198, 234)	15.9 (14.7, 17.3)	-64.3 (-69.1, -57.4)
Iraq	575 (417, 770)	78.5 (56.9, 105)	352 (239, 522)	36.8 (24.9, 54.6)	-53.2 (-71.1, -24.6)
Jordan	130 (101, 163)	94.7 (73.3, 118.2)	76 (56, 104)	31.5 (23.1, 43.5)	-66.7 (-77.8, -50.1)
Kuwait	2 (2, 3)	6 (5.3, 6.8)	3 (2, 4)	5.1 (4, 6.4)	-14.1 (-35.6, 10.4)
Lebanon	46 (33, 61)	40.5 (28.6, 53.9)	17 (12, 24)	16.3 (11.6, 22.5)	-59.8 (-74.2, -35.8)
Libya	48 (34, 66)	30.8 (22.1, 42.3)	27 (18, 39)	33.4 (22.4, 47.8)	8.4 (-37.5, 78.5)
Morocco	2562 (2038, 3148)	317.4 (252.5, 389.9)	567 (389, 890)	93.3 (63.9, 146.3)	-70.6 (-80.5, -53.2)
Oman	39 (27, 54)	58.9 (41.4, 82.1)	14 (10, 19)	18.2 (13.5, 24.1)	-69 (-80.8, -50.8)
Palestine	34 (24, 47)	35.3 (25.2, 49.4)	20 (16, 25)	16.1 (12.4, 20.2)	-54.5 (-70.7, -28.8)
Qatar	8 (6, 11)	77.2 (56.1, 101.3)	6 (4, 8)	22.8 (15.9, 31.4)	-70.5 (-81.8, -53.3)
Saudi Arabia	339 (236, 463)	62.8 (43.8, 85.9)	245 (169, 345)	53.7 (37.1, 75.9)	-14.5 (-46.6, 38.7)
Sudan	3089 (2321, 4000)	292.2 (219.5, 378.4)	2413 (1360, 3671)	200 (112.7, 304.2)	-31.6 (-61.2, 7.2)
Syrian Arab Republic	447 (323, 584)	86.5 (62.6, 113)	50 (34, 72)	21.4 (14.7, 30.8)	-75.2 (-84.4, -60.9)
Tunisia	184 (145, 230)	76.3 (59.9, 95.3)	55 (35, 79)	33 (20.8, 47.3)	-56.8 (-74, -32.3)
Turkey	1543 (1218, 1911)	91.5 (72.2, 113.3)	284 (210, 383)	29 (21.4, 39.1)	-68.3 (-78, -53.3)
United Arab Emirates	11 (8, 16)	24.1 (16.6, 33.7)	12 (7, 18)	20.8 (12.3, 32)	-13.8 (-51.4, 44.9)
Yemen	1596 (895, 2404)	234.1 (131.3, 352.6)	1705 (951, 2486)	179.7 (100.3, 261.9)	-23.3 (-58.7, 40.8)

MMR, maternal mortality ratio.

**Figure 2:**
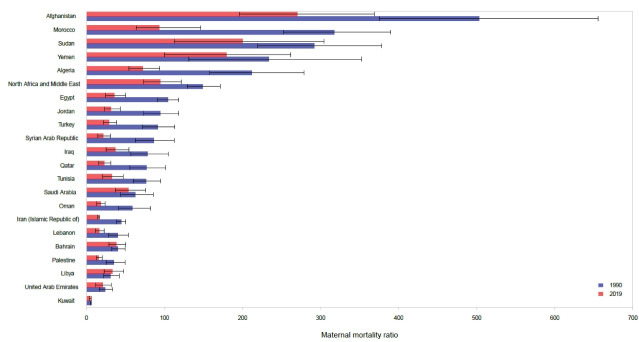
Maternal Mortality Ratio Across Countries of the Region in 1990 and 2019.

 In 1990, MMR ranged from 6.0 (5.3–6.8) in Kuwait to 502.9 (375.2–655.3) per 100 000 live births in Afghanistan. Respective figures for 2019 were 5.1 (4.0–6.4) in Kuwait to 269.9 (195.8–368.6) per 100 000 live births in Afghanistan (Figure 2). The highest percent decrease in MMR from 1990 to 2019 was observed in Syrian Arab Republic [-75.2% (-84.4, -60.9)], which mostly occurred before 2010. Actually the MMR in Syrian Arab republic increased from 18.0 (13.9, 23.0) per 100 000 live births in 2010 to 21.4 (14.7, 30.8) per 100 000 live births in 2019. The lowest percent decrease occurred in Bahrain [-5.2 (-31.7, 33.0)]. Libya was the only country in which MMR increased by 8.4% (-37.5, 78.5) from 1990 to 2019. Among the 21 countries in the region, only five countries haven’t achieved the MMR of below 70 deaths per 100,000 live births in 2019: Afghanistan, Sudan, Yemen, Morocco, and Algeria (Table 1).

 Figure S2 demonstrates the age-standardized rates of DALYs across countries in 1990 and 2019. Similar to mortality, Afghanistan ranks first and Kuwait ranks last in terms of age-standardized DALY rates per 100 000 females in both 1990 and 2019.

 In 1990, the total number of maternal deaths was higher than 2000 in four countries of Sudan [3089 deaths (2321–4000), Afghanistan [2655 (1981–3460)], Morocco [2562 (2038–3148)], and Egypt [2041 (1780–2316)] and these four countries accounted for 58.2% of total maternal deaths in North Africa and Middle East (Table 1). In 2019, however, the total number of maternal deaths was highest in Afghanistan [4038 (2930, 5514)] and this country accounted for 35.1% of total deaths in the region. Sudan [2413 (1360, 3671)], Yemen [1705 (951, 2486)], and Egypt [751 (514, 1056)] ranked second to fourth in terms of death numbers in the region. These three countries accounted for another 42.3% of all deaths in North Africa and Middle East in 2019. Afghanistan and Yemen were the only countries in which the number of maternal deaths substantially increased from 1990 to 2019. The number of maternal deaths across countries is presented in Table 1. Unlike the steady decline in all other countries, the number of deaths in Afghanistan peaked to 5,020 (3826, 6605) deaths in 2008 and declined afterwards. In Yemen as well, the number of maternal deaths peaked to 2084 (1346, 2966) in 2008 and declined afterwards. Despite the decline since 2008 in both countries, the number of maternal deaths in 2019 were still higher than 1990.

**Figure 3:**
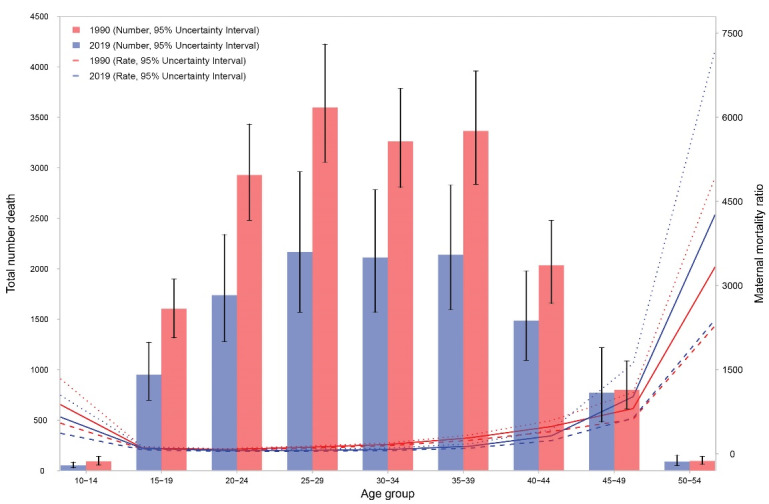
The Age Pattern of Maternal Mortality Number and Ratio in North Africa and Middle East in 2019.

 The age pattern of maternal mortality shows high numbers in middle aged women (25 to 39 years) in 2019. However, Figure 3 shows that in 2019, MMR was high in younger women (aged 10 to 14 years) and older women (aged 45 and more). The pattern was similar in 1990, though in 1990 the MMR in younger age groups was higher in than 2019 and the MMR in older age groups was lower than 2019, which probably follows the number of live births in these two age groups in 1990 and 2019. Percentages of deaths under 25 years was 26.0% in 1990 and 23.8% in 2019. The age pattern of DALY numbers and rates are demonstrated in Figure S3. Both number of DALYs and rates are high in middle-aged groups and highest in the age group of 25 to 29.

**Figure 4:**
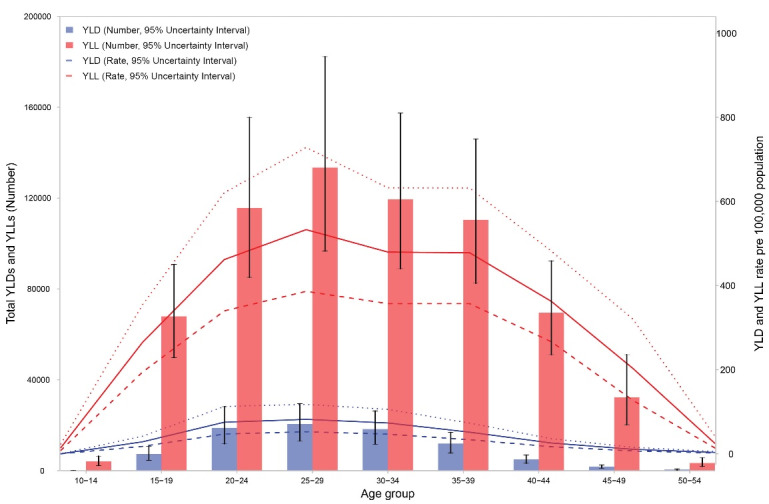
The Age Pattern of YLL and YLD Number and Age-Standardized Rates Due to Maternal Disorders in 2019.

Figure 4 shows the age pattern of YLLs and YLDs for maternal disorders. In all age groups, YLLs were higher than YLDs. YLLs and YLDs number and rates peaked in mothers aged 25 to 29 years. Age-standardized YLL rate decreased from 659.3 (573.7–759.2) per 100 000 females in 1990 to 202.8 (158.4 - 260.9) per 100 000 females in 2019. However, the change in age-standardized YLD rates was less steep, from 56.4 (38.1–77.5) per 100 000 females in 1990 to 26.0 (17.4–36.1) per 100 000 females in 2019. Figures S4 and S5 show the trend in number and age-standardized rates per 100 000 of YLLs and YLDs due to maternal disorders from 1990 to 2019. The share of YLLs out of all DALYs due to maternal disorders was 91.9% in 1990, which decreased to 88.6% in 2019, showing better prevention of maternal deaths compared to maternal disabilities. Figure S6 demonstrates the share of YLLs and YLDs out of DALYs for maternal disorders by cause in 1990 and 2019. For all causes of maternal disorders, YLLs comprise a higher proportion out of all DALYs compared to YLDs. Maternal obstructed labor and uterine rupture is an exception. YLDs account for a higher proportion of DALYs due to this disorder compared to other disorders.

**Figure 5:**
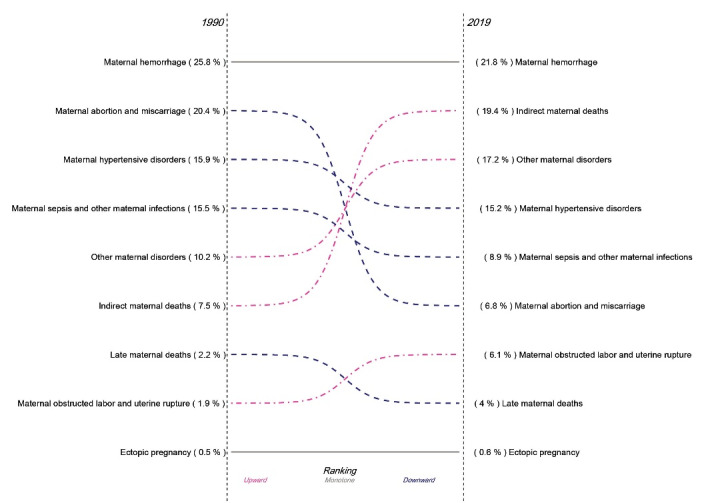
The Share of Deaths Due to Each Cause out of Deaths Due to All Maternal Disorders in North Africa and Middle East in 1990 and 2019.

 The share of each cause of death out of the total maternal deaths in North Africa and Middle East has changed between 1990 and 2019 (Figure 5). Maternal hemorrhage ranked first in both years and was responsible for over 20% of all deaths. During the time period, the share of maternal abortion and miscarriage decreased from 20.4% to 6.8%, and the share of maternal sepsis and other maternal infections decreased from 15.5% to 8.9%. There was minimal difference in the share of maternal hypertensive disorders between 1990 and 2019. On the other hand, the share of indirect maternal deaths increased from 7.5% to 19.4% between 1990 and 2019. Similarly, an increase was observed in the share of other maternal disorders from 10.2% to 17.2%. Late maternal deaths, maternal obstructed labor and uterine rupture, and ectopic pregnancy comprised 4.6% of all deaths in 1990, which increased to 7.1% in 2019.

**Figure 6:**
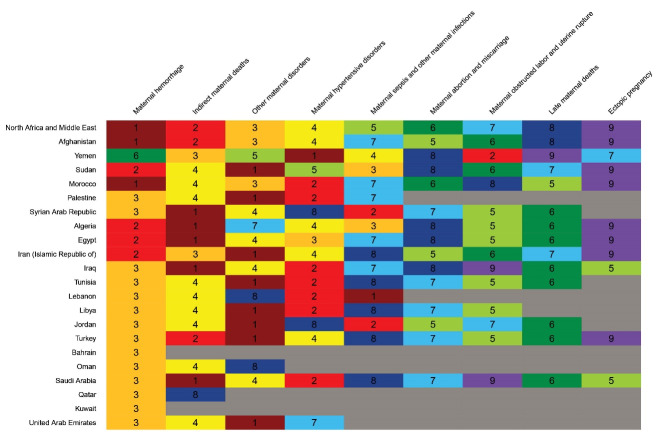
The Heatmap Showing the Ranks of Maternal Mortality by Cause Across Countries of the Regions in 2019. The gray shaded areas show no deaths. Countries are sorted based on their SDI.

Figure 6 demonstrates the order of maternal mortality causes in North Africa and Middle East and across countries in 2019. Countries are sorted based on their SDI. Maternal hemorrhage, indirect maternal deaths, and other maternal disorders rank 1^st^ to 3^rd^ in the entire region. As Afghanistan comprised a very large proportion of maternal deaths in the entire region in 2019, the order of causes in this country closely follows the order at the regional level. In 2019 in Afghanistan, a total of 31.4% of all maternal deaths were due to hemorrhage, 15.8% of deaths were due to indirect maternal deaths, 15.7% of deaths were due to other maternal disorders, 13.1% of deaths were due to maternal hypertensive disorders, 9.1% of deaths were due to maternal obstructed labor and uterine rupture, and 8.0% of deaths were due to maternal sepsis and other maternal infections.

 However, the pattern across the rest of the countries shows substantial variation. Other maternal disorders ranked first in 14 countries, most of which had high SDI. Maternal hemorrhage ranked first to third in 16 countries. Indirect maternal deaths ranked first or second in seven countries, and ranked fourth in seven other countries. Maternal hypertensive disorders ranked second to four in 18 countries. Altogether, the aforementioned four causes of mortality accounted for 73.6% of all deaths in 2019.

**Figure 7:**
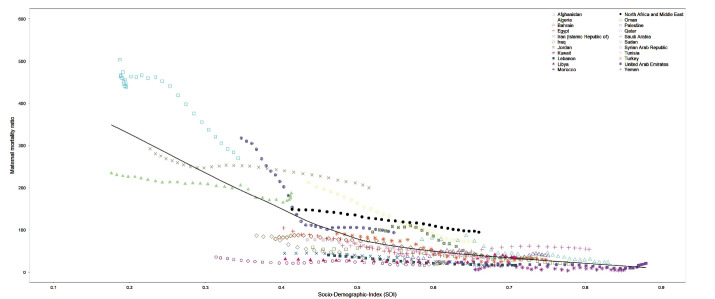
The trend in Maternal Mortality Ratio across countries along with increase in SDI from 1990 to 2019.

 Ultimately, we explored the association of SDI with MMR from 1990 to 2019 (Figure 7). There was an evident decrease in MMR along with increase in SDI from 1990 to 2019 in all countries. There was also an evident convergence in MMRs across countries implying decreasing inequality in distribution of maternal mortality since 1990. The ratio of highest to lowest MMR, in Afghanistan and Kuwait respectively, was 83.8 in 1990 and 52.9 in 2019. The same trend was observed for age-standardized DALY rates due to maternal disorders from 1990 to 2019 (Figure S7).

## Discussion

 The results of this study showed that in 2019, the MMR in the region of North Africa and Middle East [94.3 (73.4, 121.1) per 100 000 live births] was remarkably lower that the global level [145.2 (126.5, 166.8) per 100 000 live births]. Respective figures for age-standardized DALY rates in 2019 were 228.8 (183.7, 287.7) per 100 000 females in North Africa and Middle East and 324.9 (284.0, 369.1) per 100 000 females at the global level. The findings of this study demonstrated a significant decline in maternal mortality and morbidity in the region during the past three decades, though decline in mortality was more prominent than morbidity. The results of this study also demonstrated a gap between low-income and high-income countries in North Africa and Middle East. Afghanistan accounted for over one third of all maternal deaths in the region.^18^ Afghanistan, Yemen, and other low-income countries located in North Africa accounted for most of the burden imposed by maternal disorders at the regional level, while high-income Arab countries have minimal contribution to the regional burden.^19^ Maternal health heavily depends on the socio-economic status of nations and the health system infrastructure. Results also demonstrated that despite catastrophic conflicts and wars in the region, SDI has been increasing in all nations along with decrease in MMR since 1990, showing convergence and reduction in disparity. Results additionally showed that only five countries out of 21 did not achieve the SDG 3.1 target of 70 deaths per 100 000 live births in 2019: Afghanistan, Sudan, Yemen, Morocco, and Algeria, all of which are categorized as low-SDI countries. Our findings confirm that maternal mortality and disorders are key indicators of development in nations, are absolutely avoidable, and appropriate policies can substantially reduce their burden.^16,20^

 Although the impact of national socioeconomic status on maternal burden is well-recognized, there are other determinants that can affect the burden as well. Poverty is not the only driver for maternal disorders. There are low-income nations that benefit from effective health systems and are successful in preventing maternal mortality. In order to prevent maternal mortality and morbidity, fundamental changes should be made not only in resource allocation, but also in the structure of health services delivery and even non-health infrastructure. Inter-sectoral collaboration and an integrated approach towards reducing the burden of maternal disorders are essential to ensure the success of health systems.^20^ Intrapartum care strategy encompassing skilled birth attendance is key for preventing maternal mortality.^21^ Timely access to family planning programs and antenatal care, safe abortion, and postpartum care is essential for a health care system to be efficient.^20,21^ Universal health coverage can remarkably enhance the cost effectiveness and equity of the health systems.^20^ On the other hand, there are certain “patient factors” that affect access to health care. Patient factors are faulty actions of patients such as non-arrival or delayed arrival at a health facility, failure to seek legal abortion or interference with pregnancy, nonuse of prenatal care, cultural practices against utilizing required timely health care, and transportation problems.^22^ Evidence shows that women’s literacy and their financial and legal authority enhances their adherence to family planning programs, reproductive healthcare services, antenatal care, and postpartum care.^1^ It is the mission of the health care systems to ensure universal access to timely and high-quality health services and to overcome the barriers posed by both the health system and by patients.

 The age pattern of maternal mortality demonstrated large numbers of deaths in middle aged females and large rates in young and old females. There was a decline in proportion of maternal deaths below 25 years of age, which is the results of decline in total fertility rates and in fertility rate among adolescent and young girls less than 25 years of age.^23,24^ Higher MMR in age boundaries suggests that pregnancies should be planned in best age groups, which seems to be between 20 to 29 years old.

 The leading causes of maternal mortality in North Africa and Middle East have shifted since 1990. Maternal hemorrhage ranks first in 1990 and 2019, which is compatible with previous evidence.^25^ However, the share of indirect maternal deaths and other maternal disorders out of the total maternal deaths increased. Meanwhile the share of maternal abortion and miscarriage, maternal hypertensive disorders, and maternal sepsis and other infections decreased. These results demonstrated that apart from maternal hemorrhage, which ranks first in all years and mostly occurs in Afghanistan, the general share of the direct pregnancy and delivery-related complications has decreased and indirect complications prevail in 2019. This finding implies a fundamental improvement in intrapartum care strategies in the region,^21^ while post-partum hemorrhage remains the main cause of mortality in the region in general and in Afghanistan in particular.^26^

 For many years, North Africa and Middle East region has been afflicted by ongoing conflicts and wars, which has detrimentally affected the health status of the most vulnerable population, mainly women and children.^27^ Outbreaks of diseases have been occurring in many parts of the region and particularly in countries afflicted by conflict.^7^ Degradation of the health systems in affected countries poses a serious challenge to maintaining the health of mothers. Despite all of these drawbacks, maternal health has improved in all nations during the past 3 decades, but the gap between countries still exists.^7^

 To the best of our knowledge, the current study is the first that addresses maternal mortality and morbidity by cause and by country in North Africa and Middle East during the past three decades. However, our study has certain limitations as well. The main limitation of GBD studies is lack of adequate and reliable data by time and location. Second, there is still no definitive solution for estimating the interaction of HIV and pregnancy in death and we have very probably underestimated the effect of HIV on maternal mortality. HIV has been described by some sources as a risk factor for late maternal death. If this description is true, these deaths might not be captured appropriately, because neither reproductive health surveys nor demographic and health surveys quantify late maternal death. Third, due to lack of data it was not possible to estimate the contribution of infections other than HIV. Finally, we have estimated UIs for each component of the analysis. CODEm provides confirmation that the UIs for the maternal mortality model have a data coverage of 97.9%, so they could be slightly overestimated. Ultimately, limited data in countries afflicted by war, such as Syrian Arab Republic, may have led to underestimation of the burden due to maternal disorders.

 Future research should be focused on exploring the determinants of maternal disorders such as malnutrition, and the medical and non-medical cost of care for maternal disorders at national level in North Africa and Middle East.^28,29^ Future policies should be focused on enhancing the quality and quantity of existing data on levels and trends of the burden due to maternal disorders at national and regional burden. Future policies should additionally aim at ensuring timely access to antenatal care, skilled birth attendance, postnatal care, emergency obstetric care, and reproductive health care.

 In conclusion,there has been a considerable decline in maternal mortality in all countries in the region during the past three decades (except for Lybia). Inequality between countries has substantially decreased. However, there are still substantial disparities between high-income and low-income countries in terms of maternal mortality and morbidity. There hasn’t been any significant improvement in maternal disability. Prevention of maternal mortality and morbidity is a human right, which requires robust financing systems in developing countries. Reaching peace in the region can substantively improve the socio-economic status of countries and the efficiency of health systems and pave the way towards achievement of SDG goals. Yet, worryingly the COVID-19 pandemic has caused major disruptions to health services, which may negatively affect maternal health specifically among vulnerable populations.

## Supplementary Files

Supplementary file 1 contains Tables S1-S3 and Figures S1-S7.
