# Supplementary material for: Levels and Trends of Maternal Mortality and Morbidity by Cause in North Africa and Middle East, 1990 to 2019: An Analysis for the Global Burden of Disease Study 2019
Source: Arch Iran Med. 2022 Oct 1;25(10):666–75. doi: 10.34172/aim.2022.105 (PMC10685875; doi:10.34172/aim.2022.105)
Supplement: Supplementary file 1 — contains Tables S1-S3 and Figures S1-S7. [file aim-25-666-s001.pdf]

Arch Iran Med. October 2022;25(10):666-675
10.34172/aim.2022.105

Levels and Trends of Maternal Mortality and Morbidity by Cause in North Africa and Middle East, 1990 to 2019: An Analysis for the Global Burden of Disease Study 2019

Sadaf G. Sepanlou, MD, MPH, PhD[1]; Hossein Rezaei Aliabadi, MSc[2]; Reza Malekzadeh, MD[1*]; Mohsen Naghavi, MD[3*]; GBD Maternal Middle East Collaborators[#]

[1]Digestive Disease Research Institute, Tehran University of Medical Sciences, Tehran, Iran
[2]Bam University of Medical Sciences, Bam, Iran
[3]Institute for Health Metrics and Evaluation, School of Medicine, University of Washington, Seattle, USA

[#]A full list of authors is provided at the end of the paper.

Supplementary Table 1. Covariates used in CODEm models of overall maternal mortality

Supplementary Table 2. Covariates used in generation of ensemble stage 1 predictions of cause-specific maternal mortality ST-GPR models.

Supplementary Table 3. All-age DALY counts and age-standardized DALY rates per 100,000 due to maternal disorders in 1990 and 2019 and the percent change in rates during this time period.

Supplementary Figure 1. The trend in all-age number and age-standardised DALY rates per 100,000 due to maternal disorders from 1990 to 2019 in north Africa and Middle East

Supplementary Figure 2: Age-standardized DALY rates due to maternal disorders per 100,000 across countries in north Africa and Middle East in 1990 and 2019

Supplementary Figure 3. The age pattern of DALY numbers and rates per 100,000 due to maternal disorders in 2019.

Supplementary Figure 4. The trend in YLL number and age-standardized rates per 100,000 due to maternal disorders from 1990 to 2019.

Supplementary Figure 5. The trend in YLD number and age-standardized rates per 100,000 due to maternal disorders from 1990 to 2019.

Supplementary Figure 6. The share of YLLs and YLDs out of DALYs for maternal disorders by cause in 1990 and 2019

Supplementary Figure 7. The trend in age-standardized DALY rates due to all maternal disorders across countries along with increase in SDI from 1990 to 2019.

Supplementary Table 1. Covariates used in CODEm models of overall maternal mortality

Level	Covariate	Direction
**Level 1**	Age-specific fertility rate	+
	Total fertility rate (log-transformed)	+
	Maternal education (years per capita)	–
	In-facility delivery (proportion)	–
	Skilled birth attendance (proportion)	–
	Neonatal mortality ratio (log-transformed)	+
	Age-specific HIV mortality in females 10-54 (log-transformed)	+
**Level 2**	Antenatal care 1-visit coverage (proportion)	-
	Antenatal care 4-visits coverage (proportion)	-
	Age-standardised wasting (weight-for-height) summary exposure value (SEV)	+
	Age-standardised stunting (height-for-age) SEV	+
	Healthcare Access and Quality Index	-
	Age- and sex-specific SEV for high body-mass index (BMI)	+
	Age- and sex-specific SEV for high blood pressure (SBP)	+
	Underweight women of reproductive age	+
**Level 3**	Socio-demographic Index	-
	Mortality shock (cumulative rate in last 10 years)	+
	LDI (log-transformed)	-
	Hospital beds (per 1,000 population)	–

Supplementary Table 2. Covariates used in generation of ensemble stage 1 predictions of cause-specific maternal mortality ST-GPR models.

Maternal sub-cause	Country-level covariates	Direction
Maternal haemorrhage	In-facility delivery (proportion)	-
	Skilled birth attendance (proportion)	-
	Age- and sex-specific SEV for unsafe sanitation	+
	Neonatal mortality ratio (log-transformed)	+
	Maternal education	-
	Healthcare Access and Quality Index	-
Maternal hypertensive disorders	Age- and sex-specific SEV for fasting plasma glucose (FPG)	+
	Age- and sex-specific SEV for high body-mass index (BMI)	+
	Age- and sex-specific SEV for high blood pressure (SBP)	+
	Neonatal mortality ratio (log-transformed)	+
	Hospital beds (per 1000 population)	-
	Antenatal care 1-visit coverage (proportion)	-
	Antenatal care 4-visits coverage (proportion)	-
	Healthcare Access and Quality Index	-
Obstructed labour and uterine rupture	In-facility delivery (proportion)	-
	Skilled birth attendance (proportion)	-
	Underweight women of reproductive age	+

	Neonatal mortality ratio (log-transformed)<br>Hospital beds (per 1000 population)<br>Age-standardised wasting (weight-for-height) SEV<br>Age-standardised stunting (height-for-age) SEV	+<br>-<br>+<br>+
Abortion and miscarriage	Abortion legality<br>Antenatal care 1-visit coverage (proportion)<br>Antenatal care 4-visits coverage (proportion)<br>Hospital beds (per 1,000 population)<br>Maternal education<br>Healthcare Access and Quality Index	-<br>-<br>-<br>-<br>-<br>-
Ectopic pregnancy	Abortion legality<br>Pelvic inflammatory disease age-standardised prevalence<br>Antenatal care 1-visit coverage (proportion)<br>Antenatal care 4-visits coverage (proportion)<br>Hospital beds (per 1,000 population)<br>Maternal education<br>Healthcare Access and Quality Index	-<br>+<br>-<br>-<br>-<br>-<br>-
Maternal sepsis and other maternal infections	In-facility delivery (proportion)<br>Skilled birth attendance (proportion)<br>Age- and sex-specific SEV for unsafe sanitation<br>Age- and sex-specific SEV for fasting plasma glucose (FPG)<br>Antenatal care 1-visit coverage (proportion)<br>Antenatal care 4-visits coverage (proportion)<br>LDI (log-transformed)<br>Healthcare Access and Quality Index	-<br>-<br>+<br>+<br>-<br>-<br>-<br>-
Other maternal deaths	In-facility delivery (proportion)<br>Skilled birth attendance (proportion)<br>Antenatal care 1-visit coverage (proportion)<br>Antenatal care 4-visits coverage (proportion)<br>LDI (log-transformed)<br>Age- and sex-specific SEV for high body-mass index (BMI)<br>Maternal education<br>Healthcare Access and Quality Index	-<br>-<br>-<br>-<br>-<br>+<br>-<br>-

Indirect maternal deaths	In-facility delivery (proportion)<br>Skilled birth attendance (proportion)<br>Antenatal care 1-visit coverage (proportion)<br>Antenatal care 4-visits coverage (proportion)<br>LDI (log-transformed)<br>Age- and sex-specific SEV for high body-mass index (BMI)<br>Maternal education<br>Healthcare Access and Quality Index	-<br>-<br>-<br>-<br>-<br>+<br>-<br>-

Supplementary Table 3. All-age DALY counts and age-standardized DALY rates per 100,000 due to maternal disorders in 1990 and 2019 and the percent change in rates during this time period.

	All-age DALY counts (95% UI) 1990	Age standardized DALY rates (95% UI) 1990	All-age DALY counts (95% UI) 2019	Age standardized DALY rates (95% UI) 2019	Percent change from 1990 to 2019
North Africa and Middle East	1120074 (986199, 1275046)	715.6 (630.4, 817.7)	740462 (593259, 930918)	228.8 (183.7, 287.7)	-68 (-73.9, -60.4)
Afghanistan	157850 (120198, 201543)	3429.8 (2587.9, 4425.6)	242898 (179597, 326264)	1478.9 (1089.7, 1974.8)	-56.9 (-68.2, -39.7)
Algeria	102457 (77389, 132425)	849.5 (640.4, 1099)	41945 (32465, 53223)	182.5 (141.5, 229.7)	-78.5 (-85, -68.8)
Bahrain	410 (335, 489)	170.1 (140.6, 202.3)	349 (278, 440)	52.4 (41.4, 66.4)	-69.2 (-76.5, -59.6)
Egypt	128415 (112393, 145121)	483.7 (423.1, 546.8)	53240 (38633, 70654)	102.7 (74.4, 136.8)	-78.8 (-84.7, -71.5)
Iran (Islamic Republic of)	55572 (48579, 63072)	210.4 (184.6, 237.3)	17789 (15308, 20766)	36.9 (31.7, 43.3)	-82.5 (-84.9, -79.7)
Iraq	36719 (27349, 47659)	497.8 (370.7, 649.7)	24604 (17633, 34312)	110.6 (79.7, 154.6)	-77.8 (-85.2, -66.5)
Jordan	7932 (6257, 9802)	535.8 (421.3, 663.1)	5347 (4112, 6992)	93.2 (71.6, 123.1)	-82.6 (-87.6, -75.2)
Kuwait	294 (231, 369)	32.3 (25.8, 40.3)	388 (292, 501)	13.2 (9.9, 17.2)	-59 (-64.7, -52.9)
Lebanon	3266 (2466, 4180)	210.8 (158, 270.9)	1348 (997, 1764)	49.8 (37.5, 64.8)	-76.4 (-83.7, -66)
Libya	3284	212.9 (158.9, 279.6)	1866	44.9 (32.7, 60.7)	-78.9 (-86.5, -68.5)

	(2451, 4330)		(1353, 2523)		
Morocco	151925 (121512, 185650)	1222 (982.6, 1491.4)	35173 (24979, 53259)	181.1 (128.8, 275)	-85.2 (-89.8, -77.4)
Oman	2568 (1898, 3442)	388.7 (286.7, 522.1)	1127 (878, 1419)	55.1 (43.1, 68.8)	-85.8 (-90.3, -79.3)
Palestine	2364 (1773, 3153)	286.7 (211.4, 382.2)	1672 (1321, 2093)	65.6 (52.2, 81.9)	-77.1 (-84.1, -67.8)
Qatar	540 (412, 695)	317.2 (243.4, 404.5)	510 (375, 664)	51.2 (38.3, 65.9)	-83.9 (-88.8, -77.3)
Saudi Arabia	21186 (15257, 28197)	339.5 (243.9, 453.2)	15373 (11306, 21024)	72.4 (53.3, 97.1)	-78.7 (-85.8, -67)
Sudan	196162 (151232, 248621)	2170.7 (1683.8, 2748.6)	149901 (90961, 222562)	723.7 (433, 1069.7)	-66.7 (-79.9, -49.3)
Syrian Arab Republic	29341 (22196, 37623)	524.7 (395.8, 672.7)	3877 (2827, 5142)	51.2 (37, 68.6)	-90.2 (-93.4, -85.6)
Tunisia	12282 (9937, 15144)	287.2 (234.1, 353)	3887 (2636, 5332)	62.7 (42.5, 86.8)	-78.2 (-85.7, -67.7)
Turkey	100877 (81121, 121543)	336.7 (269.2, 406)	22849 (17818, 29451)	50.9 (39.7, 65.8)	-84.9 (-88.8, -79.7)
United Arab Emirates	867 (633, 1137)	122.5 (90.8, 161.1)	936 (656, 1271)	24.5 (17.5, 32.7)	-80 (-85.9, -71.8)
Yemen	105008 (61103, 153806)	1952.1 (1184.6, 2809)	114631 (70418, 160621)	724.5 (448.4, 1016.9)	-62.9 (-77.9, -38.8)

UI: Uncertainty Interval

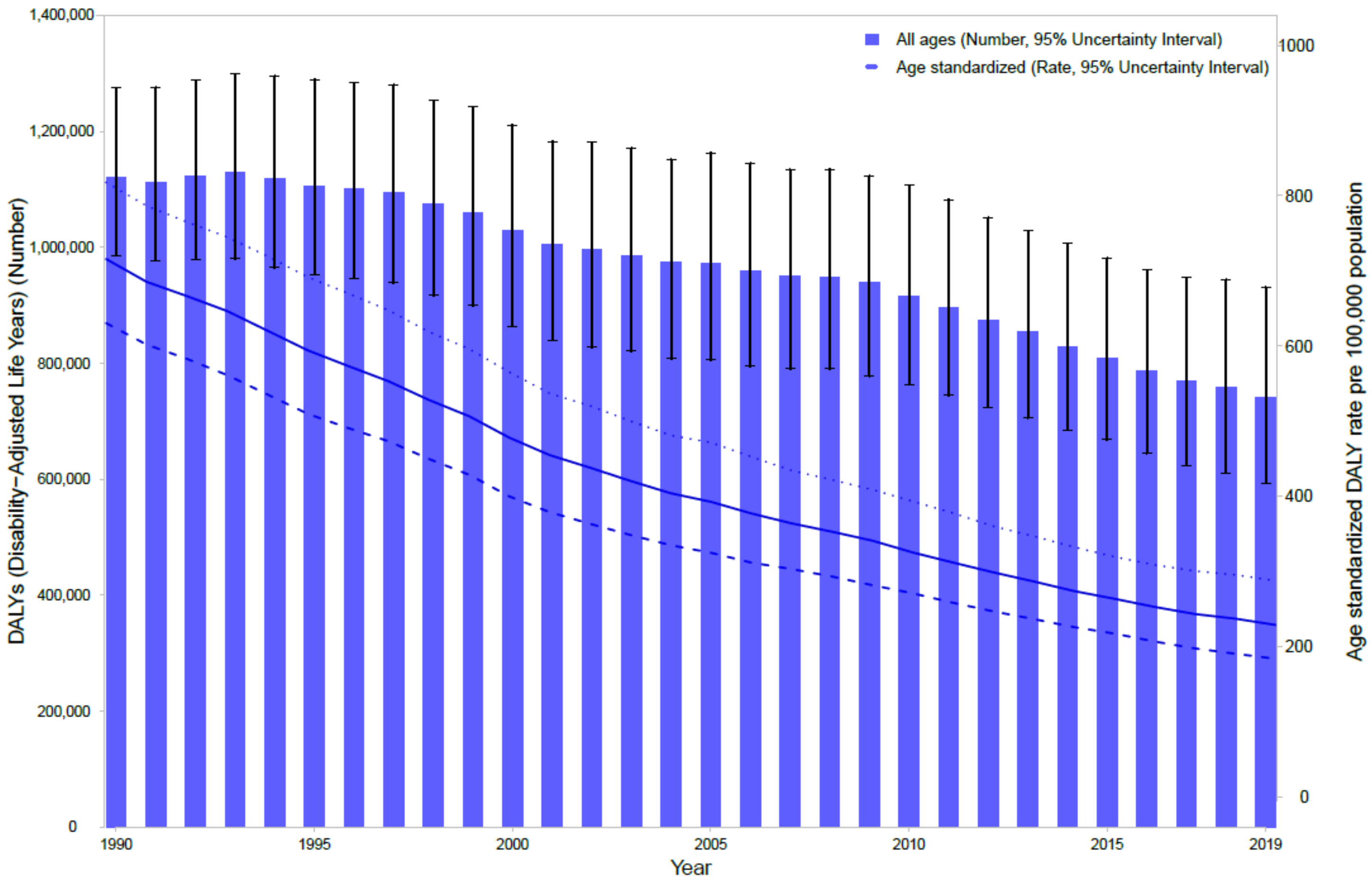

Supplementary Figure 1. The trend in all-age number and age-standardised DALY rates per 100,000 due to maternal disorders from 1990 to 2019 in north Africa and Middle East

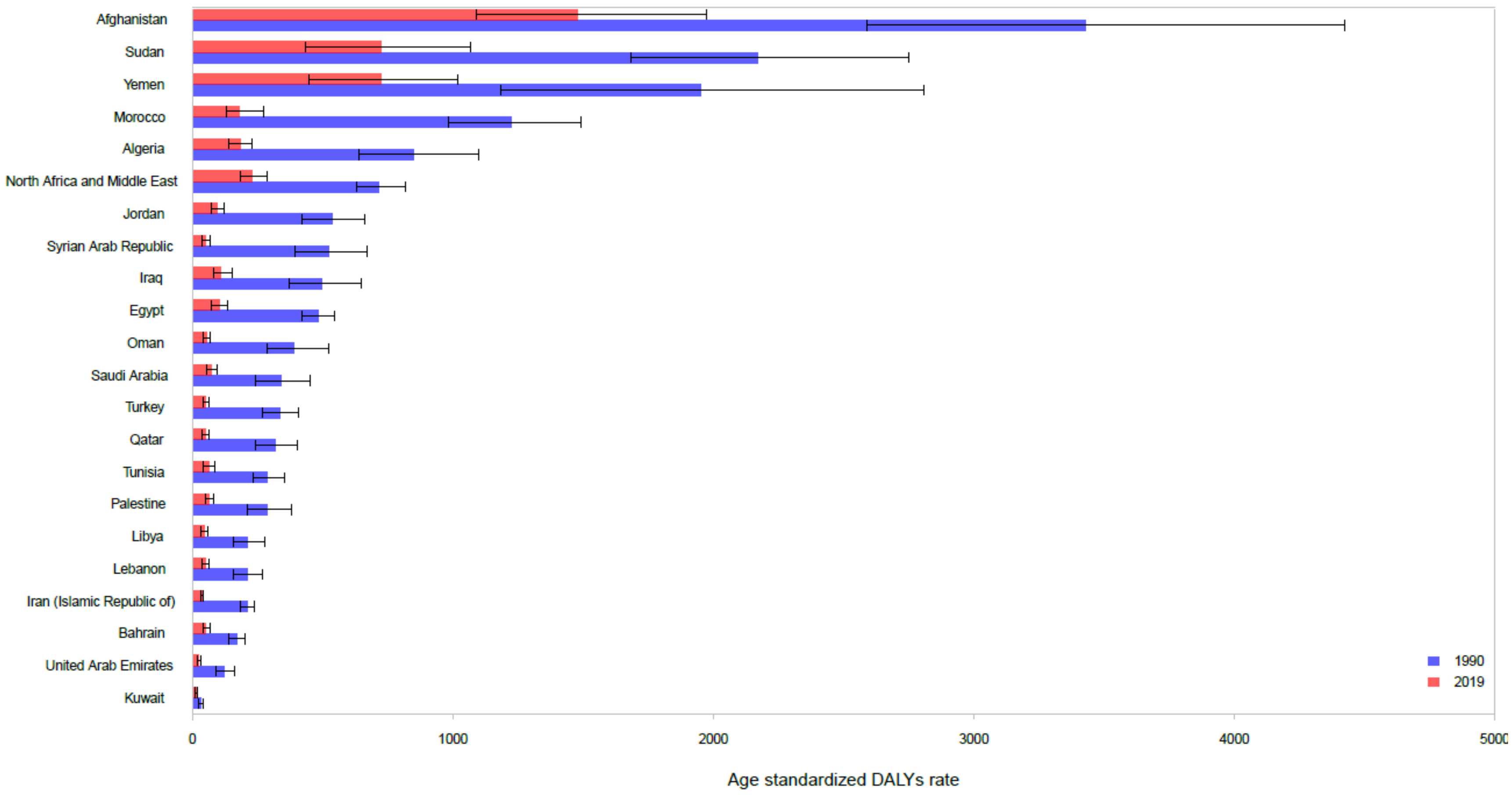

Supplementary Figure 2. Age-standardized DALY rates due to maternal disorders per 100,000 across countries in north Africa and Middle East in 1990 and 2019

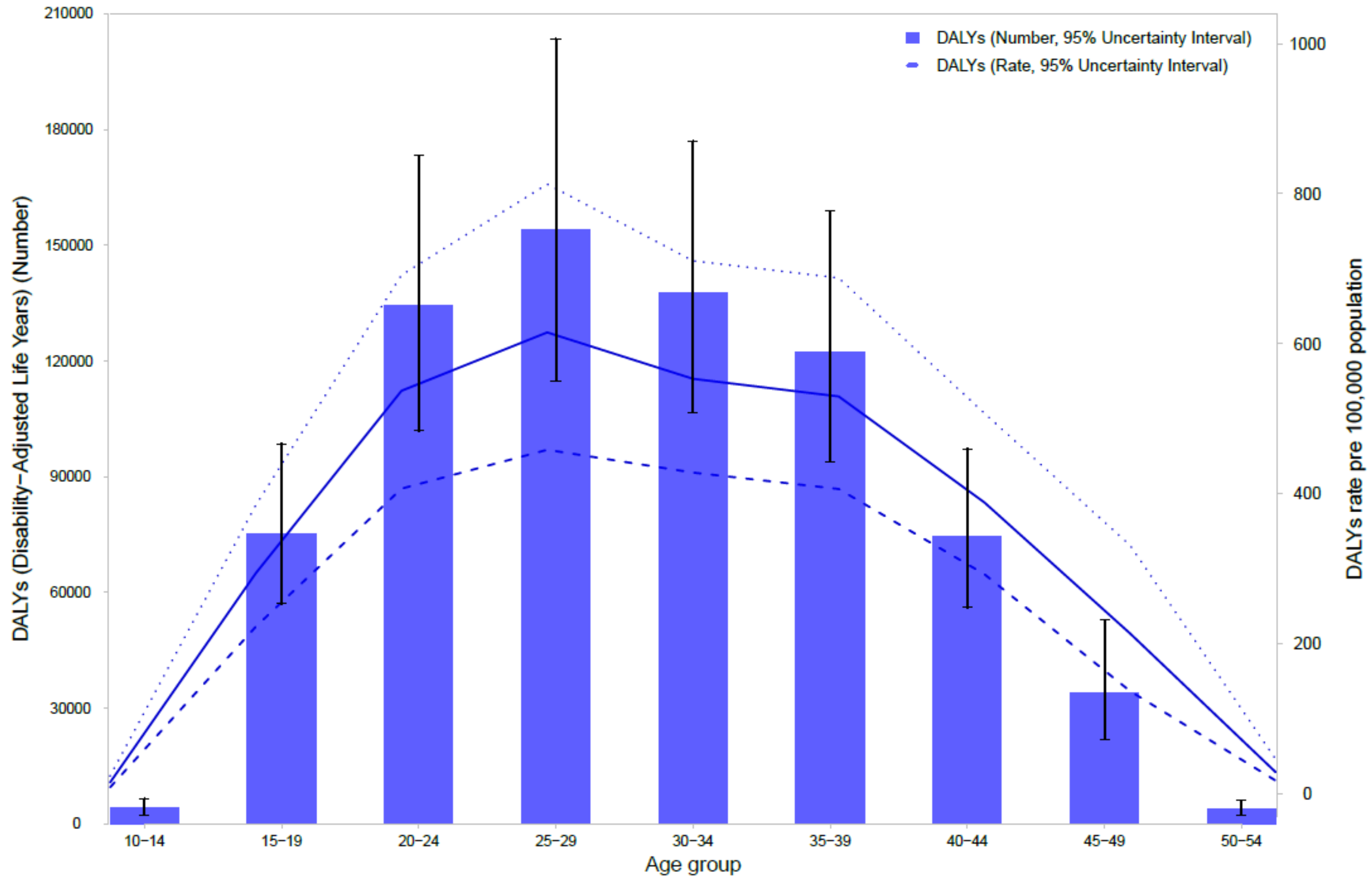

Supplementary Figure 3. The age pattern of DALY numbers and rates per 100,000 due to maternal disorders in 2019.

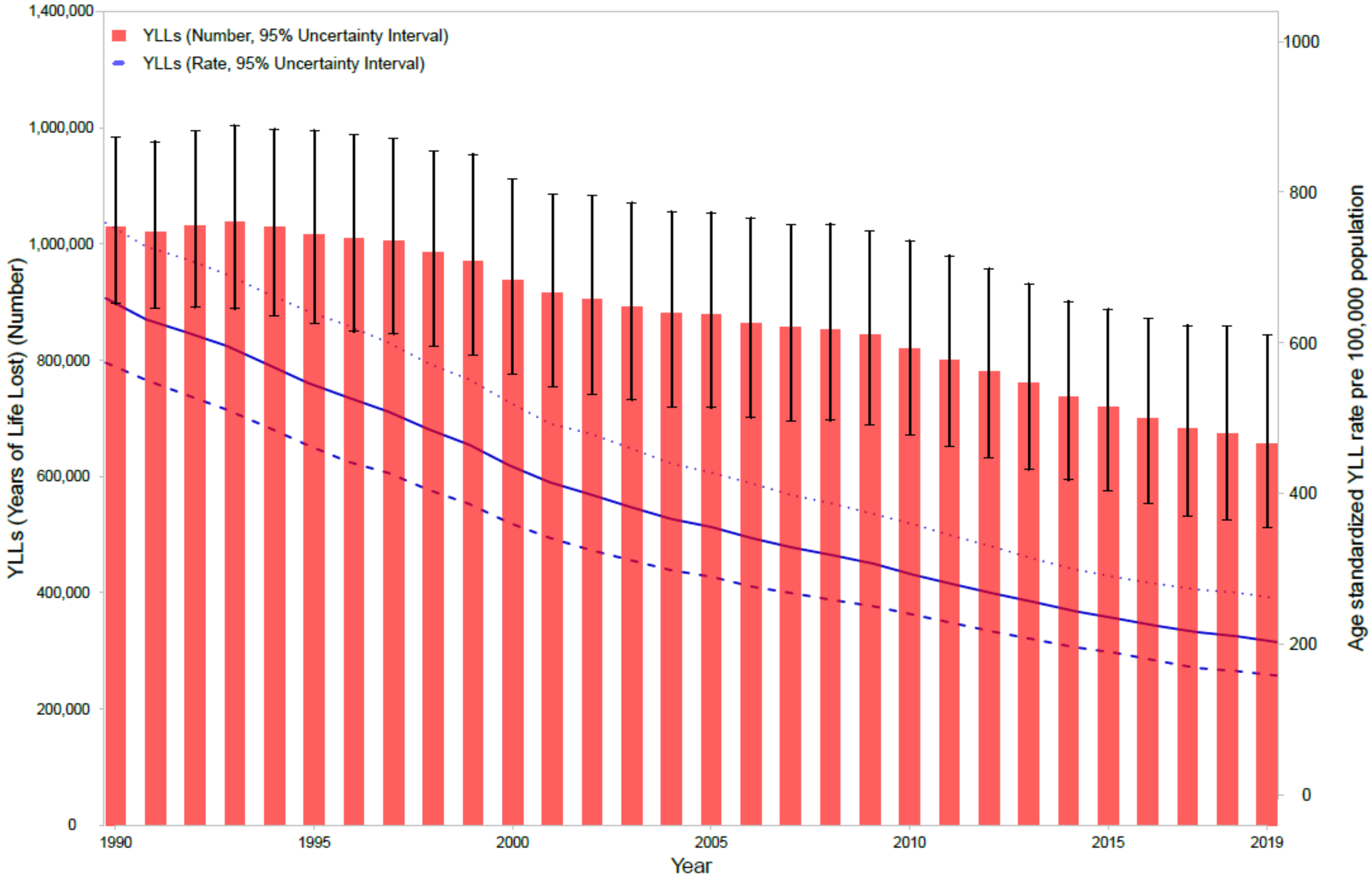

Supplementary Figure 4. The trend in YLL number and age-standardized rates per 100,000 due to maternal disorders from 1990 to 2019.

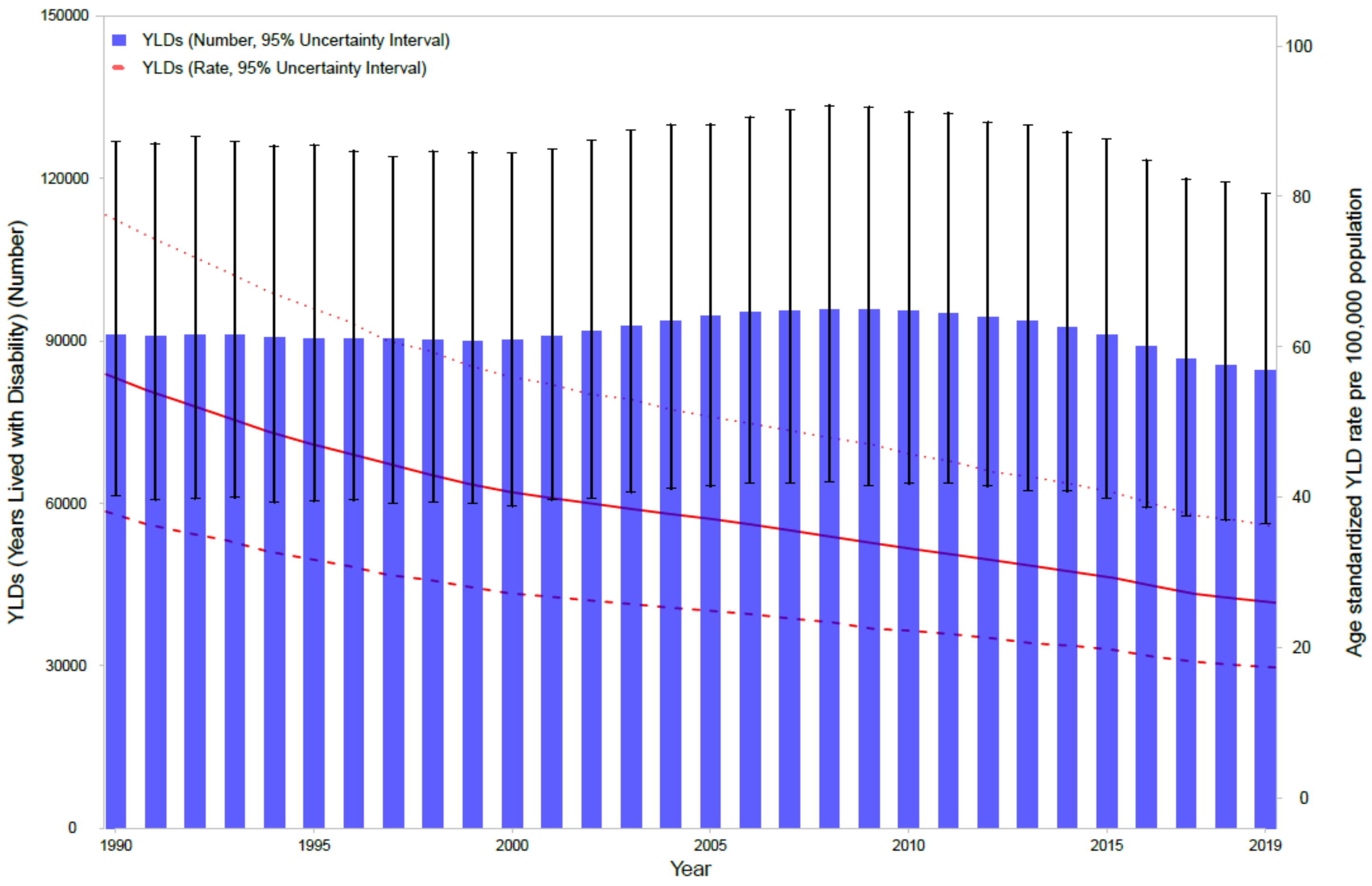

Supplementary Figure 5. The trend in YLD number and age-standardized rates per 100,000 due to maternal disorders from 1990 to 2019.

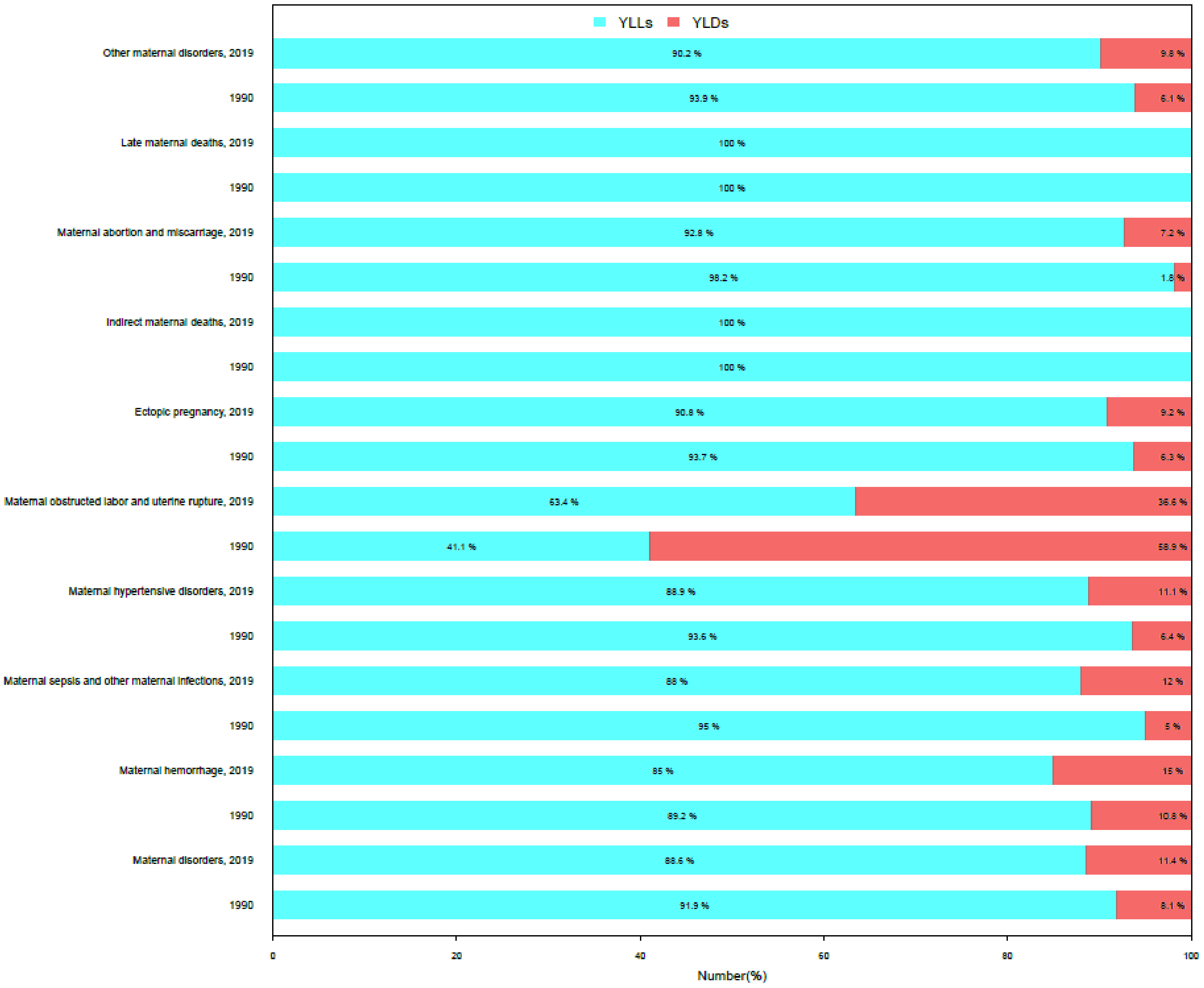

Supplementary Figure 6. The share of YLLs and YLDs out of DALYs for maternal disorders by cause in 1990 and 2019

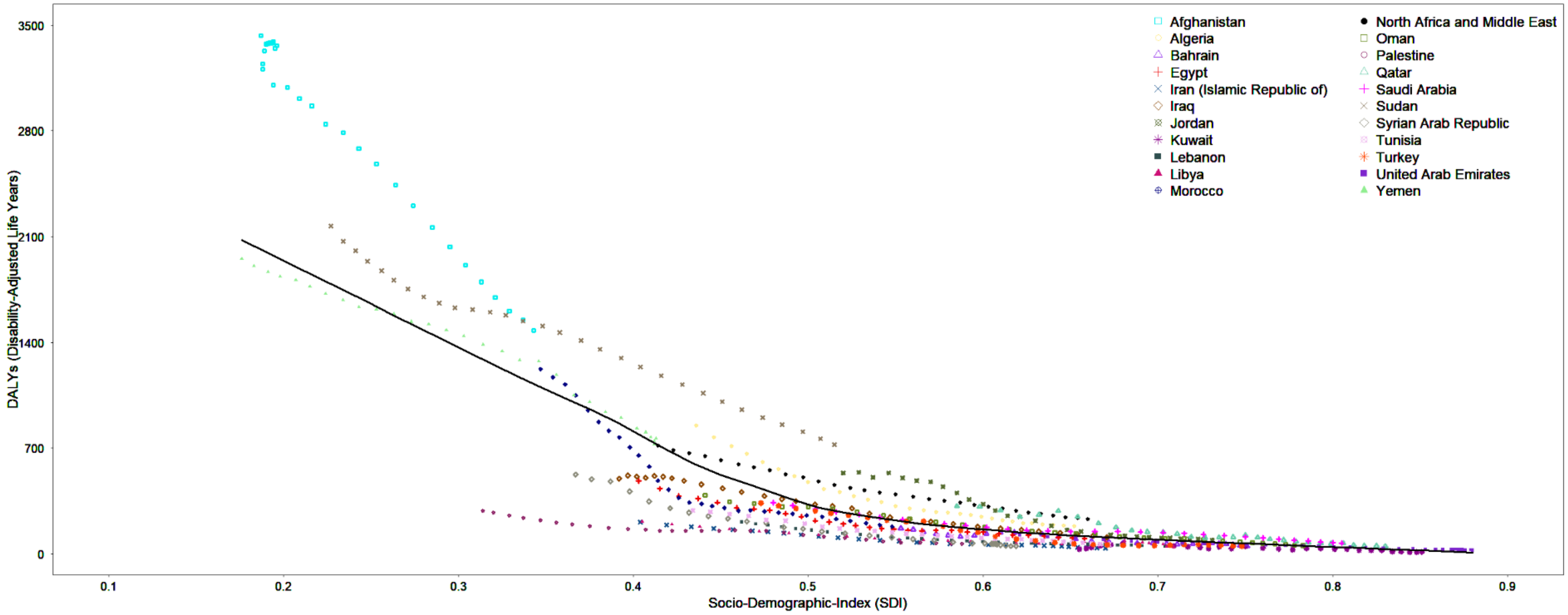

Supplementary Figure 7. The trend in age-standardized DALY rates due to all maternal disorders across countries along with increase in SDI from 1990 to 2019.
